# Routine Immunization - Do People Know About It? A Study Among Caretakers of Children Attending Pulse Polio Immunization in East Delhi

**DOI:** 10.4103/0970-0218.39240

**Published:** 2008-01

**Authors:** Rahul Sharma, Sanjiv K Bhasin

**Affiliations:** Department of Community Medicine, VMMC and Safdarjung Hospital, New Delhi, India; 1 Department of Community Medicine, UCMS and GTB Hospital, Delhi - 110 095, India

**Keywords:** Care takers, knowledge, routine immunization

## Abstract

**Research question:**

Do caretakers of children under five years have sufficient knowledge regarding routine immunization (RI)?

**Objective:**

To assess the knowledge about RI among caretakers of young children.

**Settings:**

Pulse polio immunization centres in East Delhi.

**Study design:**

Cross-sectional study.

**Participants:**

Six hundred and eighty-two caretakers accompanying children under 5 years to pulse polio booths in November 2006.

**Study tool:**

Pre-tested semi-open-ended questionnaire.

**Statistical analysis:**

Proportions, Chi-square test.

**Results:**

The proportions of respondents who had awareness about different aspects of RI, such as weekday of RI (37.0%), age group for RI (49.1%), number of visits required in the first year of life (27.0%), were all low. When asked to name the four diseases covered under the RI program in Delhi, only 268 (39.3%) could name at least three. The education level of respondents was strongly associated with their knowledge about RI.

**Conclusion:**

The need of the hour is to make RI a ‘felt need’ of the community. Making caretakers more aware about RI is a vital step in achieving this goal.

## Introduction

The goal of immunizing children against chief diseases responsible for child mortality and morbidity is indeed a noble one. However, it is not an easy task to achieve. In a developing country like India, the sheer logistics of the numbers of the target population that stretches across geographically diverse regions make universal immunization of children a Herculean task. However, the health sector of this country is making admirable achievements in that several millions of potential life years have been saved from getting lost to vaccine preventable diseases through the universal immunization program (UIP).

There are several reasons to aim for universal coverage. The factors that should be helpful are many. The Indian culture promotes safe nurturing of children. Hardly do we find parents who risk their children to life-threatening diseases, unless they being unaware or misinformed. All vaccines under the routine immunization programme are provided free-of-charge. However, the figures for the coverage of routine immunization (RI) are lagging. The current level of coverage of ‘fully-immunized’ children under the national immunization programme is quite low, as pointed out by several studies.(1–8)

So what could be the possible hindrances that hamper progress? The main reasons identified for poor coverage include the inadequacy of community participation in RI and information, education and communication (IEC) activities.(2) The importance of knowledge/awareness about RI as a factor for its success is brought out by previous studies that “not aware of the needs of vaccination” is the main reason for children not being fully immunized.(2,4) This study was carried out to assess how much do the people motivated enough to get their under-five children for pulse polio vaccination on an Intensive Pulse Polio Immunization Day, know about various aspects of routine immunization (RI).

## Materials and Methods

It was a community-based cross-sectional study undertaken in November 2006. The respondents were people who took children under five years to a pulse polio immunization booth in the national capital territory of Delhi on 12^th^ November. Undergraduate medical students of our institute were deputed at the polio booths for this purpose. Each student was asked to interview 20 people at the booth where s(he) was deputed, it being convenience sampling. The anonymity of the respondents was assured and their verbal consent was taken. The study being a students' project was reviewed and approved by the department experts.

A semi-open-ended questionnaire was developed by one of the authors (RS) under consultation with experts from the department. The questionnaire was pre-tested and suitably modified prior to use. It was used to record the responses of the interviewees to various questions asked to test their knowledge about RI. The aspects inquired about included the weekday and age for RI, diseases covered, number of visits required and the newer vaccines being introduced in Delhi. The responses were fed into a computer-based spreadsheet. Out of the 40 students who were deputed, three did not submit their forms, while those of two were rejected due to inadequate data. Of the forms submitted, three had interviewed less than 20 people each. The final sample analyzed consisted of 682 people. Proportions of responses to various questions regarding RI were calculated. Chi-square tests were used to analyze the association between knowledge status and various socio-demographic variables.

## Results

The time when the respondent came to the center was recorded. Most of the interviews (65.7%) had been conducted within the 3 h of activity by 12 p.m. The age of 682 respondents ranged between 11 and 77 years, the median age being 30 years. Nearly two-thirds (67.0%) of them belonged to the age group of 21-40 years, while 13.3% were below 21 years and 19.6% were above 40 years. There was almost equal representation of both genders (50.4% males and 49.6% females). While 95 (13.9%) persons were illiterate, 15.8% had finished primary school, 39.6% had done middle or high schooling, and 209 (30.6%) were graduates.

The relationships of the respondents to the children being accompanied are depicted in Table 1. Children were mostly accompanied by mothers (31.2%) or fathers (32.8%). The age of the child was also recorded. While 170 children (24.9%) were infants, the remaining 512 were between the ages of 1 and 5 years. The respondents were asked which day of the week RI of under-five children is carried out in government facilities. Universally, Wednesday is designated as the day of RI in Delhi, with some facilities extending it to one or more additional days too. So, credit was given if a respondent answered Wednesday. Only 252 (37.0%) respondents correctly knew the day of RI, 176 (25.8%) gave a wrong answer while the remaining 37.2% gave no answer.

**Table 1:** Relationships of the respondents with the underfive children

Relation	Number	Percentage
Mother	213	31.2
Father	224	32.8
Grandparent	73	10.7
Brother	33	4.8
Sister	27	4.0
Others	93	13.6
Neighbour	19	2.8
Total	682	100.0

Less than half (49.1%) knew that RI is done for children up to the age of 5 years. The respondents were asked to mention how many times a child has to be taken for RI in the first year of life. The question was contextual as IEC campaigns including television spots publicizing the same have started being run under the NRHM. While 180 (27.0%) respondents correctly mentioned the number of visits as ‘four’, 50.4% gave a wrong answer and 22.6% did not know the answer.

A question was put to the respondents to name the four diseases for which vaccines are administered under the RI programme in Delhi (typhoid and hepatitis B are not a part of national immunization program yet). The respondents were asked to mention whether vaccines for these diseases are available as part of RI. The maximum respondents knew about measles (61.0%) followed by tuberculosis (52.5%). Less than one in two respondents knew about the availability of hepatitis B and typhoid vaccines. While 268 (39.3%) knew at least three of the four diseases covered by the RI programme, only 23.9% knew all four diseases. One hundred and twenty-two (17.9%) persons believed that vaccines for none of the diseases are part of the RI programme.

Only 123 (18.0%) respondents correctly mentioned the age for measles vaccination as 9 months. Nearly half the respondents (45.3%) just said ‘don't know’ or ‘cannot say’. Since typhoid vaccine is administered between 2 and 5 years of age, any age in that range was taken as a right answer. Still, only 247 (36.2%) could mention the age correctly. A question was put as to which disease is the hepatitis B vaccine administered for. The students were briefed to accept ‘jaundice’, ‘liver’ or ‘*peelia*’ (Hindi word for jaundice) as a correct response. While 246 (36.1%) knew it correctly and 165 gave a wrong answer, the majority of respondents (39.7%) offered no answer. The results are summarized in Table 2.

**Table 2:** Distribution of the study population with respect to their knowledge status about routine immunization

Study question	Knew correctly	Knew incorrectly	Did not answer
Day of RI in the week	252 (37.0)	176 (25.8)	254 (37.2)
Age group for RI	335 (49.1)	180 (26.4)	167 (24.5)
Number of visits	184 (27.0)	344 (50.4)	154 (22.6)
required for RI in the first year of life			
Diseases for which vaccines available in RI		
Tuberculosis	358 (52.5)	137 (20.1)	187 (27.4)
Measles	416 (61.0)	113 (16.6)	153 (22.4)
Hepatitis B	327 (47.9)	168 (24.6)	187 (27.4)
Typhoid	319 (46.8)	165 (24.2)	198 (29.0)
Age at which measles vaccine given	123 (18.0)	250 (36.7)	309 (45.3)
Age at which typhoid vaccine given	247 (36.2)	98 (14.4)	337 (49.4)
What is hepatitis B vaccine given for	246 (36.1)	165 (24.2)	271 (39.7)

RI = routine immunization, Figures in parentheses are in percentage

A clear difference regarding the knowledge status about various aspects of RI was observed among mothers, fathers and other relations [Table 3]. Mothers were significantly more likely to be aware about the day of RI in the week, the number of visits required in infancy and the diseases covered under the RI programme in Delhi. Another variable that was found to have an association with the knowledge about RI was the education level of the respondents. As shown in Table 4, illiterate respondents were least likely to be sentient about RI, while higher proportions of those who had attained graduation or higher education knew significantly about RI. No significant difference was observed between the ‘early-comers’ (those who had come before 12 p.m.) and the ‘late-comers’ with respect to the knowledge level about RI. Respondents who accompanied infants were more likely to know the correct age of measles vaccination (*P* = 0.004) while those who accompanied 1-5-year olds were more likely to know the correct age of typhoid vaccination (*P* = 0.078).

**Table 3:** Difference in knowledge status among mothers, fathers and other relations about RI (figures are proportions who had correct knowledge)

Study question	Mothers (*n* = 213) (%)	Fathers (*n* = 224) (%)	Others (*n* = 245) (%)	*P*-value for difference
Day of RI in the week	45.5	34.8	31.4	0.006
Age group for RI	51.2	50.4	46.1	0.497
Number of visits required for RI in the first year of life	33.3	27.7	20.8	0.010
Knew names of at least three diseases covered by RI*	47.9	42.0	29.4	<0.001

* Tuberculosis, measles, hepatitis B and typhoid were asked about.

**Table 4:** Association of education level with knowledge status about routine immunization (*n* = 682 for each question)

Study question	Illiterates (*n* = 95) (%)	Primary/middle/high school (*n* = 378) (%)	Graduates (*n* = 209) (%)	*P*-value for difference
Day of RI in the week	28.4	37.8	39.2	0.169
Age group for RI	33.7	51.1	52.6	0.005
Number of visits required for RI in the first year of life	18.9	27.5	29.7	0.14
Knew names of at least three diseases covered by RI*	27.4	39.2	45.0	0.014
Knew why hepatitis B vaccine given for	23.2	32.0	49.3	<0.001
Knew the age for measles vaccination	10.5	18.5	20.6	0.101

Proportions in each group who had correct knowledge

## Discussion

RI is important not just for polio but also for reducing the burden of mortality and morbidity due to other diseases as covered under RI. The opportunity presented by the selected cohort of under-five children being brought on the pulse polio days was utilized to study the knowledge level of the accompanying people about RI, specifically in Delhi.

Nearly two-thirds of the subjects were mothers or fathers of under-five children. It was found that the proportion of respondents who were aware about various aspects of RI was quite low. Singh *et al.* too had found in their study that mothers had fair knowledge regarding the need for immunization but had poor knowledge regarding the diseases that it prevents.(3) A study in Bangladesh found lack of knowledge in mothers on vaccine preventable diseases to be strongly associated with no or delayed immunization (odds ratio 16.7; *p*<0.0001).(9) A UNICEF report had identified ‘caregivers not knowing the value of vaccines and time and place of administration’ as the top challenge to immunization programmes worldwide.(10) A study in Rajasthan had found that specific information about vaccine preventable diseases other than polio was very limited in mothers.(8)

A declining trend in awareness about diseases covered under RI was observed, especially the age at which the vaccine is administered. The only exception was tuberculosis; the awareness about it was lower probably because we asked about ‘tuberculosis’, not BCG. However, this in itself may be a significant finding that many people do not associate BCG with the disease it is actually meant for. Previous studies have shown a declining trend in coverage, with vaccines given at higher ages having progressively lower coverage percentage.(3,4,7,11) Our finding of a declining trend of awareness levels about vaccines with the age for vaccination can be a direct contributory factor for the ‘drop-out’ in the coverage of vaccines. Our finding that knowledge about RI increases with the education level of the respondents correlates well with that of Dalal *et al*.(11)

A limitation of the study was that systematic random sampling could not be done because of logistical reasons, and this could be a potential source of bias. A point to be remembered is that the respondents comprised of caretakers who brought their children to the centers for polio immunization, and the results would not be generalizable to the whole population. It may be presumed that the knowledge and motivation levels regarding RI would be low among those who did not turn up, and thus the actual figures for the community as a whole can be expected to be lower than the study's findings.

The necessity of planned IEC activities to promote RI as a felt need of the caretakers has been highlighted earlier.(4,6,10) The utilization of peripheral level workers like ANMs and ASHAs to personally interact with stakeholders in their area and promote RI is an urgent need of the nation. In another study, 123 of 166 mothers stated interpersonal approach as the most effective way to improve the success of the existing programme.(8)

The pulse polio days should be utilized as a good opportunity for the advocacy of RI to the target audience, as recommended by Singhal *et al.* too.(12) This should be made a component in the training of health personnel being posted at the polio booths. Good ideas to promote RI can also be adopted from other countries of the world. For example, USA has a National Immunization Awareness Month (NIAM) each year to increase awareness about RI.(13)

RI of all children has been long recognized and credited as one of the most cost-effective interventions possible by the health sector. The need of the hour is to make it a ‘felt need’ of the community. Increasing the knowledge and understanding of caretakers of young children about the essentiality and benefits of RI would be a strong step forward in achieving this goal.
